# Metagenomic detection of central nervous system infections missedby conventional testing

**DOI:** 10.1172/jci.insight.189295

**Published:** 2025-05-20

**Authors:** Noely Evangelista Ferreira, Michael G. Berg, Antonio C. da Costa, Mary A. Rodgers, Esper G. Kallas, Cassia G. Terrasani Silveira, Mateus Vailant Thomazella, Ana Carolina Soares de Oliveira, Layla Honorato, Heuder G.O. Paião, Renan Barros Domingues, Carlos Senne, Marina F. Côrtes, Tania R. Tozetto-Mendoza, Hélio R. Gomes, Maria Laura Mariano Matos, Geovani de Oliveira Ribeiro, Steven S. Witkin, Gavin A. Cloherty, Maria Cassia Mendes-Correa

**Affiliations:** 1 Departamento de Infectologia e Medicina Tropical, Faculdade de Medicina, and; 2 Laboratório de Investigação Médica LIM 52, Instituto de Medicina Tropical, Faculdade de Medicina, University of São Paulo, São Paulo, Brazil.; 3 Infectious Diseases Research, Abbott Diagnostics, Abbott Park, Illinois, USA.; 4 Senne Liquor Diagnóstico, São Paulo, Brazil.; 5 Divisão Neurologia - Hospital das Clinicas da Faculdade de Medicina da USP, University of São Paulo, São Paulo, Brazil.; 6 Faculdade de Medicina, Universidade de São Caetano do Sul, São Paulo, Brazil.; 7 Departamento de Biologia Celular, Universidade de Brasília (UNB), University of São Paulo, São Paulo, Brazil.; 8 Department of Obstetrics and Gynecology, Weill Cornell Medicine, New York, New York, USA.

**Keywords:** Clinical Research, Infectious disease, Molecular diagnosis

## Abstract

Community-acquired infectious meningoencephalitis is associated with high rates of mortality and morbidity, compounded by limited access to diagnostic resources. The current study assessed acute central nervous system (CNS) infections in patients with meningoencephalitis enrolled in a hospital-based diagnostic surveillance study in São Paulo, Brazil. Cerebrospinal fluid (CSF) was collected from 600 patients between March 2018 and November 2019 and initially screened for a broad range of pathogens according to a local diagnostic algorithm. Standard microbiological and molecular diagnostic methods were applied. Metagenomic sequencing was used as a complementary approach to investigating etiology in instances where no pathogen was initially identified. Standard testing identified infectious etiologies in 292 patients (48.6%), with 227 (77.7%) confirmed as viral infections, predominantly caused by enteroviruses (*n* = 144) and herpesviruses (*n* = 40). Nonviral agents were identified in 65 patients (22.3%). Metagenomic sequencing (mNGS) of 277 of 308 undiagnosed patients revealed several additional potential etiologies, including *Parvovirus B19*, *Toxoplasma gondii*, *Picobirnavirus*, other enterovirus species and *Vesivirus*, the latter being associated with CNS infection for the first time. These findings underscore the complexity of CNS infections and highlight the potential of metagenomics to improve diagnostic accuracy, inform treatment strategies, and support efforts to address future pandemics.

## Abstract 2

Our research focuses on the prevalence and etiology of community-acquired infectious meningoencephalitis among patients enrolled in a hospital-based surveillance study conducted in São Paulo, Brazil, from March 2018 to November 2019. Our findings underscore the significant role of enteroviruses and herpes viruses as major pathogens responsible for a majority of cases. Furthermore, we highlight the utility of metagenomic sequencing (mNGS) in uncovering additional infectious agents that are often missed by conventional diagnostic tests. mNGS significantly enhances the detection of pathogens compared to traditional methods. Specifically, mNGS identified pathogens in cases where conventional testing failed, thus broadening the understanding of CNS infections. The identification of diverse pathogens informs treatment strategies and has implications for public health, especially in managing outbreaks and potential future pandemics. The results provide valuable insights into the complexity of CNS infections within diverse populations, emphasizing the need for advanced diagnostic techniques. We presents critical insights into central nervous system infections and advocates for the adoption of advanced diagnostic techniques such as mNGS to enhance treatment outcomes and address public health challenges associated with infectious diseases.

## Introduction

Central nervous system (CNS) infections are among the most serious public health problems worldwide, culminating in high rates of mortality and morbidity. They result in significant neurological sequelae that severely affect quality of life with long-term consequences and impose a high-cost burden on the health care system (1). Worldwide, a significant portion of patients diagnosed with meningitis (15%–60%) or encephalitis (40%–70%) do not have an etiological agent identified (1). Although Brazil has accumulated information related to CNS infections, most of the studies have only analyzed small case series or only investigated specific etiologic agents. Data from the Brazilian Ministry of Health indicate a lack of reliable information regarding the etiology of most instances of infectious meningitis and encephalitis diagnosed in this country. A major barrier to identifying etiological agents in these patients is the difficulty in having access to rapid and sensitive diagnostic tests (2).

The present study aimed to determine the infectious etiology in cases of suspected acute CNS infection for individuals enrolled in a hospital-based surveillance study. After a clinical interview by either an infectious disease physician or a neurologist, and a clinical diagnosis of CNS infection, a cerebrospinal fluid (CSF) sample was collected. All CSF samples underwent testing for a range of pathogens using microbiological and molecular tests, according to a local diagnostic algorithm. A metagenomic sequencing approach was performed as a complementary diagnostic tool to determine the etiology of patients where no pathogen was identified following the initial screening.

## Results

### Pathogen identification by conventional diagnostic tests.

**Figure 1:**
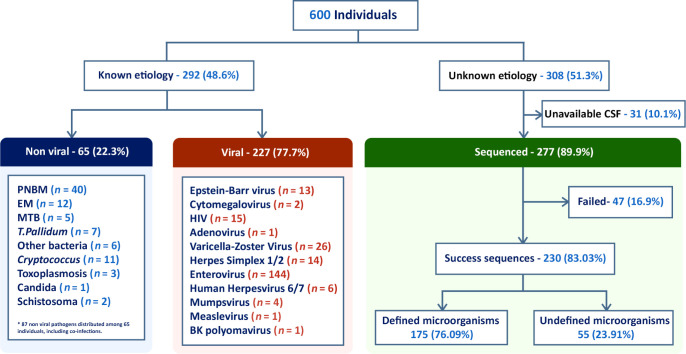
Flowchart of the study protocol in patients with acute meningitis or meningoencephalitis. The chart details patient enrollment and exclusion criteria, the distribution of diagnostic etiologies following analysis with standard microbiological tests and real-time PCR, and subsequent metagenomic analysis. PNBM, postneurosurgical bacterial meningitis; EM, epidemic meningitis; MTB, *Mycobacterium tuberculosis*; and *T*. *pallidum*, *Treponema pallidum*.

**Figure 2:**
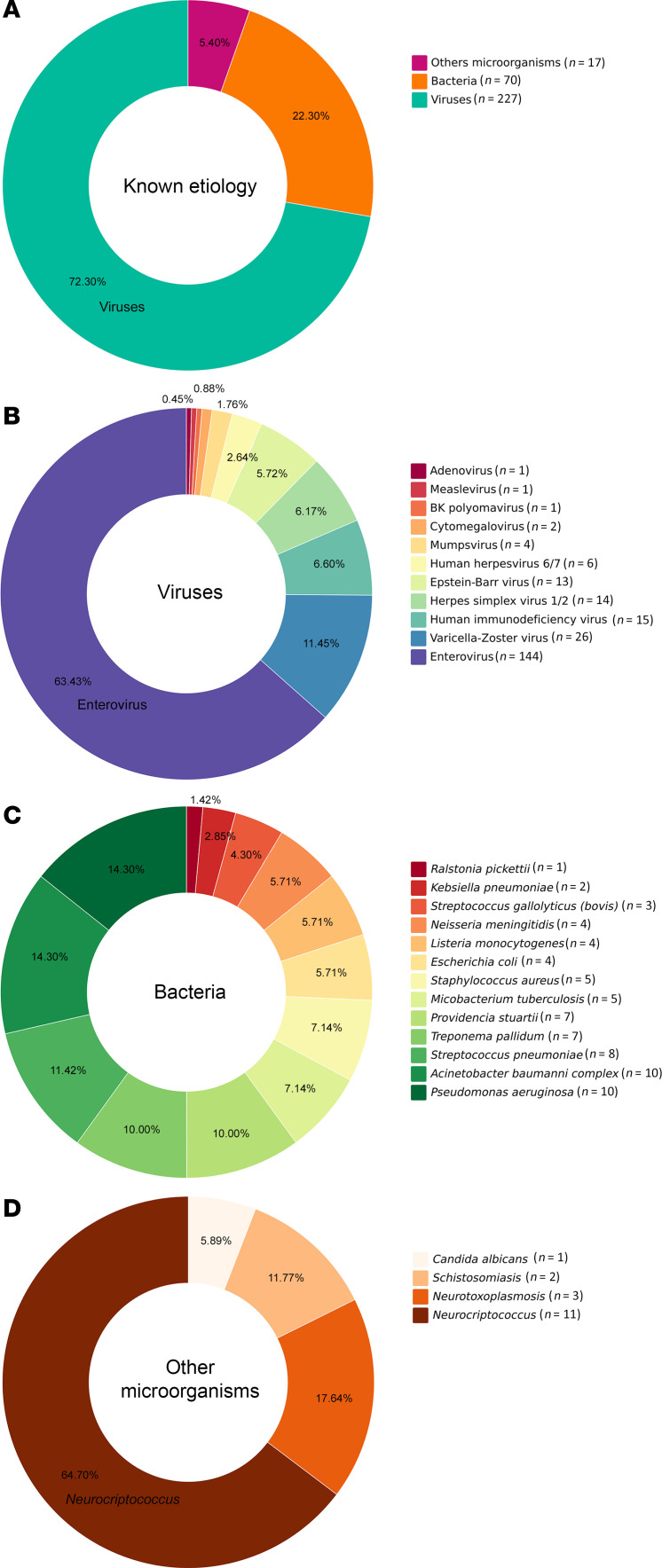
Distribution of the microorganisms detected by qPCR among 279 samples from patients with acute meningitis or meningoencephalitis. (**A**) Distribution of known etiologies including bacteria, viruses, and other microorganisms among the samples. (**B**) Detailed percentage distribution of various viruses identified. (**C**) Distribution of bacterial pathogens identified. (**D**) Composition of other microorganisms found. Each segment of the charts corresponds to the proportion of each pathogen in relation to the total identified in each category.

CSF samples from 600 patients were analyzed, 327 (54.5%) males and 273 (45.5%) females, according to the flow chart shown in Figure 1. Their ages ranged from < 1 year old to 79 years old, with 125 patients (20.8%) < 12 months, 32 from 1 to 5 years (5.3%), 124 (20.6%) from 5 to 20 years, 246 (41%) from 20 to 60 years, and 73 (12.1%) over 60 years. Standard-of-care testing (microbiological, PCR, and histology) identified infectious etiologies in 292 patients (48.6%), 227 of which (77.7%) had a confirmed viral infection (Figure 1). The most abundant causes were Enterovirus (*n* = 144) and the *Alphaherpesvirinae* subfamily (*n* = 40), the latter being HSV-1 or HSV-2 (*n* = 14) and varicella zoster virus (VZV) (*n* = 26). Other detected agents were HIV (*n* = 15), EBV (*n* = 13), human herpesvirus 6 (HHV-6) and HHV-7 (*n* = 6), mumps virus (*n* = 4), measles virus (*n* = 1), and BK virus (human polyomavirus 1) (*n* = 1), detected by PCR (Figure 2). In addition, 65 individuals (22.3%) were diagnosed with nonviral infections. A total of 87 nonviral agents, including coinfections, were confirmed in these 65 individuals (Figure 1). Different bacteria were identified by culture or PCR (Figure 2 and Table 1). Other microorganisms such as *Candida albicans*, *Cryptococcus neoformans*, *Schistosoma mansoni*, and *Toxoplasma gondii* were also identified (Figure 2 and Table 1). Seventeen of the CSFs with defined diagnosis were positive for 2 or more agents. *Herpesvirus* was the more frequent agent in association with other pathogens (Table 2).

### Metagenomic next-generation sequencing (mNGS) revealed a potential infectious etiology in previously undiagnosed meningoencephalitis subjects.

Samples with unknown etiology underwent complementary analysis through mNGS, leveraging random primer to amplify all nucleic acid and enable detection of viruses, bacteria, parasites, and fungi. Of the 308 initially undiagnosed patients, 277 were available for subsequent metagenomics analysis (Figure 1). Thirty-one samples were excluded due to insufficient CSF volume. After applying the standard filters using the DiVir pipeline, sequences considered for further analysis were obtained in 230 (83.0%) CSF samples. The total number of reads obtained were 1,887,661,726, with a median of 7,813,750 and SD of 4,636,693 (Supplemental Table 1; supplemental material available online with this article; https://doi.org/10.1172/jci.insight.189295DS1).

**Figure 3:**
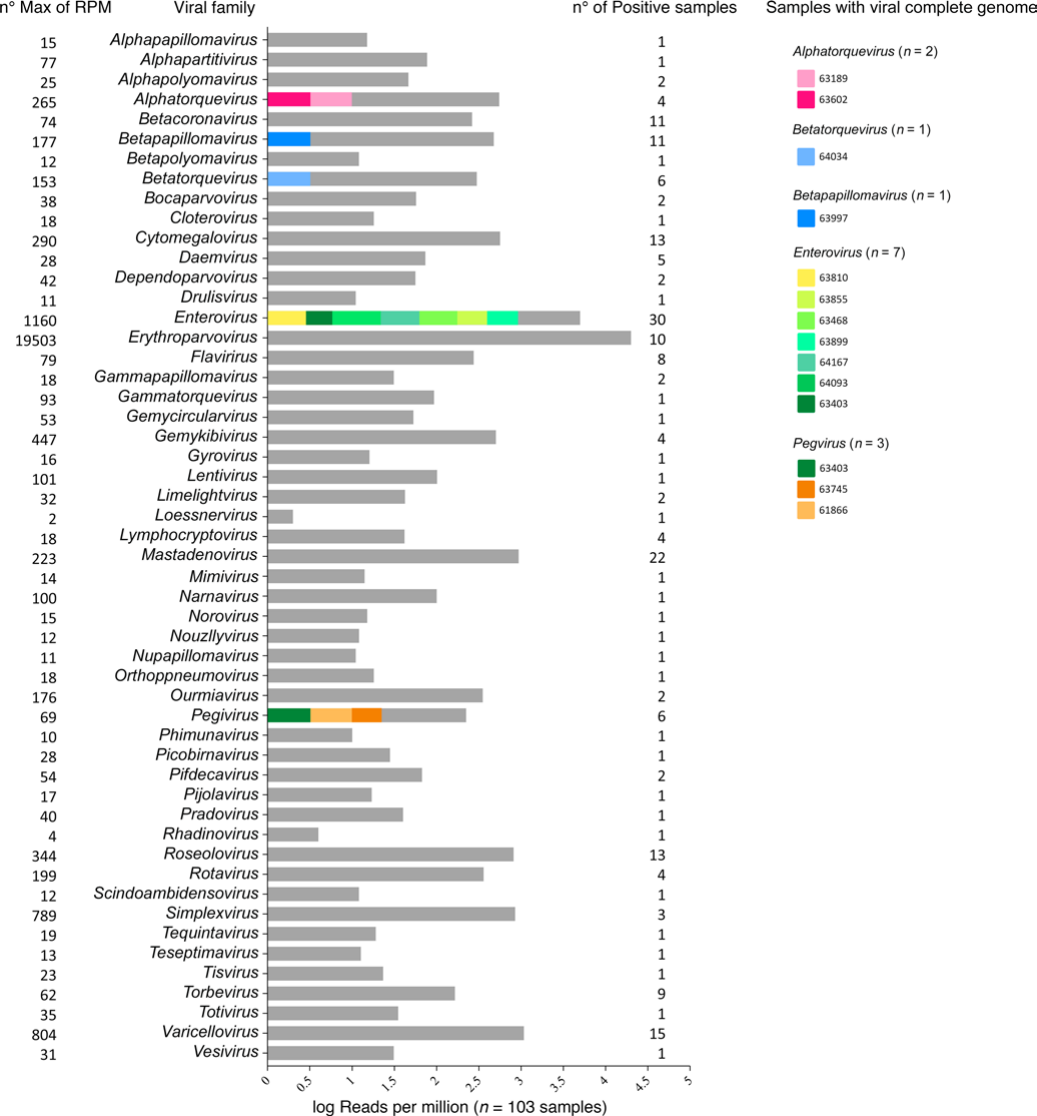
Distribution of viral genera detected by mNGS in cerebrospinal fluid (CSF) samples. Distribution of viral genera identified in cerebrospinal fluid (CSF) samples, based on reads per million (*n* = 103). Each genus is represented by different colors, indicating whether it represents a complete or near-complete genome (color) or fragments of genome (gray). The color coding for each genus is displayed on the right side of the histogram, showing the identity of the samples associated with each viral genus. No. max of RPM indicates the maximum number of reads per million (shown on the left), while the number of positive samples is indicated on the right.

mNGS analysis revealed the presence of microorganisms from different taxons: archea, bacteria, eukarya, phage, and virus (Supplemental Figure 1). Viruses were identified in 103 of the samples analyzed (Figure 3). For Group 1, the complete or near-full genome of specific viruses, with confirmatory PCR, were obtained in 14 patients (Table 3). Among these were additional enteroviruses (*n* = 7) and *Parvovirus B19* (*n* = 1) patients that were missed by conventional PCR. *Enterovirus* infections included *Enterovirus* A (*Coxsackie* A6) and a variety of *Enterovirus* B/*Echovirus*. Reads were classified as belonging to *Echovirus* E18, E11, *Coxsackie* B3, and numerous related species. The individual with *Parvovirus B19* presented with B19 encephalopathy in a newborn (3). The full genomes of Torque teno virus (TTV; *n* = 8) and human pegivirus-1 (*Pegivirus*; *n* = 6) were also found in the CSF of several patients. These were confirmed by using PCR (Supplemental Table 2).

Three Group 2 patients are noteworthy from a clinical perspective. The first was a patient with a diagnosis of acute myeloid leukemia, under immunosuppressive therapy, who presented with acute onset of fever, dysarthria, and expression aphasia. CSF analysis results were negative for all agents tested including *Toxoplasma gondii* by PCR. Cranial MRI was inconclusive. At this time, we received and stored a CSF sample at our laboratory. One week after the onset of symptoms, a new lumbar puncture was performed, and at this time, the CSF evaluation tested positive for toxoplasmosis by PCR. Metagenomic analysis of the first CSF sample, initially deemed negative by PCR, also detected the presence of *T*. *gondii*. For this patient, we note that mNGS detected Toxoplasma in the CSF, 1 week prior to PCR.

**Figure 4:**
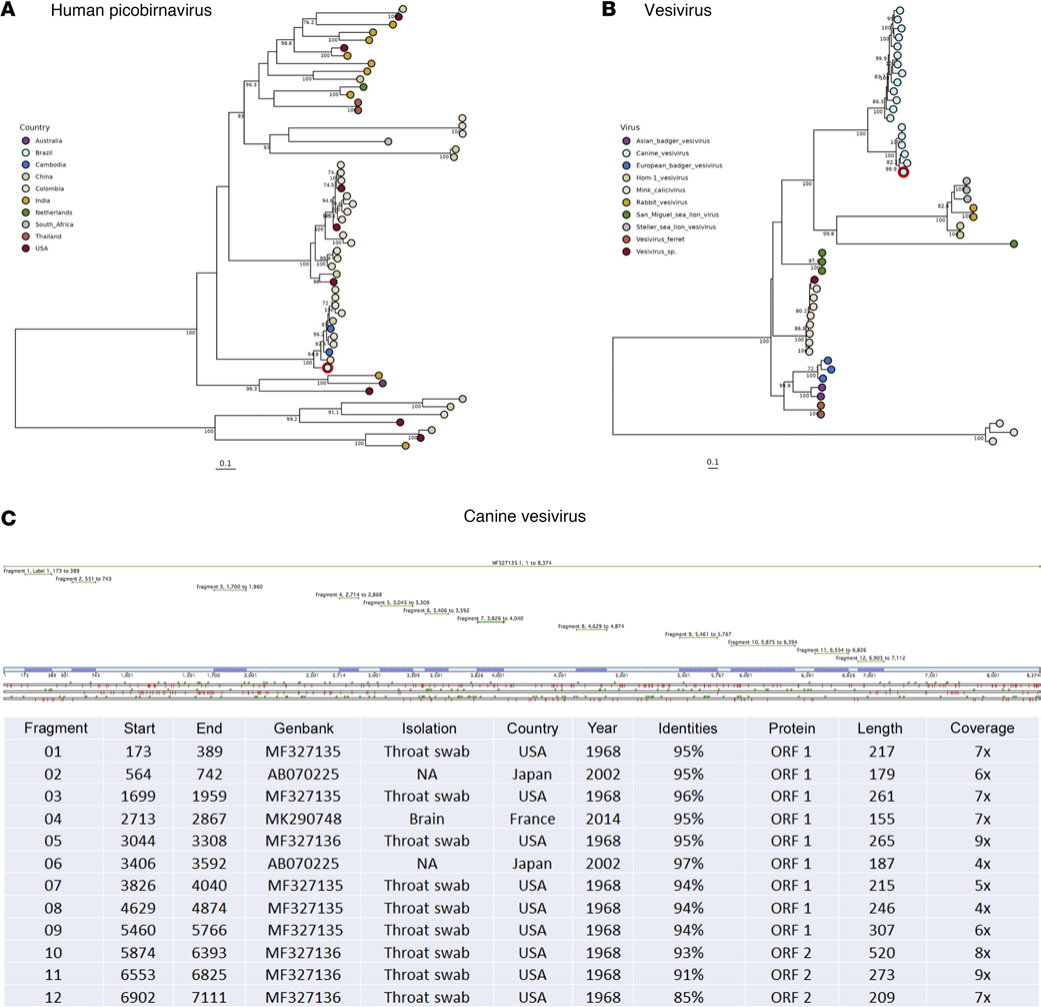
Phylogenetic analysis and genomic fragment details of picobirnaviruses and vesiviruses. (**A**) Maximum Likelihood tree for *Picobirnavirus* based on the RdRp region, with samples annotated and colored according to geographical location. The genome from this study is highlighted in red. (**B**) Maximum likelihood tree of vesiviruses based on a partial genome sequence, annotated with virus species colors, and with this study’s genome highlighted in red. (**C**) Detailed distribution of 12 genomic fragments of *Vesivirus* detected in cerebrospinal fluid samples, including key genomic details and sample history spanning from 1968 to 2014 across the USA and Japan.

The second patient was a young child with polio-like symptoms and acute neurologic sequelae. After multiple workups yielded no etiology, mNGS detected only *Picobirnavirus* in the CSF. While PBV is typically associated with gastrointestinal symptoms, we note that 10 days before admission to the hospital, the mother indicated the child had an acute respiratory illness (ARI). Basic Local Alignment Search Tool (BLAST) analysis of the RNA-dependent RNA polymerase (RdRp) sequence showed high identity to genotype 3 strains previously linked to ARI in South America and Asia (4) but not previously reported in Brazil (Figure 4A). The third was an unusual instance of a 58-year-old woman with fever, mental confusion, and dysarthria who, after multiple workups, yielded no etiology; mNGS detected a *Vesivirus* (30% coverage; Figure 4, B and C), which had not previously been identified in CSF from a patient with neurologic involvement.

For Group 2 viral candidates, we obtained partial genome coverage in patients where PCR was positive, negative, or not attempted. Beginning with agents known to cause encephalitis, there were additional patients (*n* = 15) of *Enterovirus* B infections (Figure 3). These were deemed positive according to our cutoff criteria. However, it is likely they were missed by BioFire and Neuro 9 kits due to low viral loads corresponding to the low number of reads obtained by mNGS. Similarly, we detected 15 instances of VZV with low coverage (Figure 3). Three unexpected viruses are worth noting: human adenovirus A31 (12% coverage), *Bocaparvovirus primate2* (11% coverage), and primate *Gemykibivirus* (50% coverage) (5).

Two individuals were positive for parasites. *Taenia solium* is a porcine tapeworm and common cause of neurocysticercosis. It infects the nervous system and causes epileptic seizures. Reads for this helminth (and related species) were found in a patient who presented with fever and seizures. This individual had a previous diagnosis of neurocysticercosis and was presented at our hospital with an acute onset of fever and seizures. After multiple workups yielded no etiology, mNGS detected sequences of 5s and 28s ribosomal RNA from *Taenia solium* in the CSF, which confirmed previously diagnosed neurocysticercosis. For fungi, we observed patients elevated for filamentous fungi like *Cladophialophora* and *Cladosporium* genus, known to be neurotropic and cause cerebral abscesses and meningitis. We also identified different samples enriched for the *Candida* genus. *Alternaria alternata* was also identified in 1 individual. In Group 3, different microorganisms, such as bacteriophages, fungi, bacteria, and viruses, were classified: *Alternaria* spp*.*, *Bocaparvovirus primate*, *Gemykibivirus*, and *Diutina rugosa*. Many of these microorganisms are ubiquitous in the environment.

## Discussion

Determining the etiology of CNS infections is challenging, with approximately 50%–70% of encephalitis and meningitis patients going without a diagnosis (1). According to the Brazilian Ministry of Health, there is scarce information regarding infectious etiology in most instances of meningitis and encephalitis in this country (2). Identifying the involved pathogen is vital for both therapy and prognosis (1). In the present study, enteroviruses and herpesviruses were the most frequently detected viral causes. *Herpesvirus* infections are extremely common in the general population. Their seroprevalence ranges from 60% to 90% (6). In their acute or chronic phase, these viruses can induce a range of neurological manifestations. Alternatively, they can remain in a state of latency in nervous system tissue (7). The mechanisms associated with their reactivation are not fully understood but appear to be multifactorial and vary among the different species. Considering these factors, interpretations of the consequences of *Herpesvirus* detection in CSF of patients with meningitis or encephalitis should be conducted with great caution. It is possible that the *Herpesvirus* is a true copathogen associated with the observed clinical neurological manifestations, and the current pathology could be the result of the association between 2 infectious agents concomitantly. Alternatively, the identification of herpesviruses could represent the presence of a latent form of the virus in the CNS without a specific pathogenic role. It is important to highlight that, after a standard diagnostic approach, 17 individuals presented with more than 1 microorganism identified in CSF (Table 2). Among them, in 16 instances of multiple organism detection, a virus from the herpes group was identified in association with other pathogens. According to medical records, in none of these patients was the *Herpesvirus* identified considered as the main cause of the neurological symptoms. 

VZV was the most common herpesvirus, accounting for 26 of the 40 identified instances. Our results support previous research that indicates a high occurrence of VZV in the CNS (8). Recent studies have described new circulating VZV clades in Brazil, suggesting that migration patterns may contribute to the frequency of VZV patients identified in this country (9). It is important to highlight that the actual number of detected VZV infections in Brazil is likely underestimated because only severe instances are required to be reported, per Brazilian Ministry of Health guidelines (10). These findings reinforce the inclusion of VZV analysis during clinical investigation of acute CNS infections in Brazil.

Interestingly, HIV was identified in 15 patients included in our study who were predominantly males (9 of 15) with ages between 2 and 75 years. HIV was accompanied by other pathogens in 7 patients (HHV-6, enterovirus, and VZV), and previous HIV infection was only confirmed in 3 of these individuals. The absence of more complete clinical data regarding the other patients prevents us from making specific comments on these patients. They could involve chronic or acute instances of HIV infection. Our data could serve as a warning for the presence of acute HIV infection as one of the agents of viral meningitis in the community, perhaps less identified as such, due to the difficulty of access to specific HIV diagnostic tests in CSF.

We demonstrated the utility of mNGS to identify potential pathogens in the CSF in patients where previous standard testing was negative. mNGS has been utilized in a variety of different research and clinical situations, including diagnosing meningoencephalitis. Indeed, in teaching hospital and research settings, mNGS procedures have received CLIA certification, and the results reported to physicians are used to aid diagnosis (8). The unbiased nature of mNGS allows amplification of any pathogen (e.g., viral, fungal, etc.), and sensitivity continues to improve. This sequence-agnostic and culture-independent method helps us to identify how microorganisms are circulating and how they are evolving, 2 fundamental steps to ensure pandemic preparedness and prevention (6, 9). In our study, a substantial diversity of microbial and viral entities was identified. Among samples in which a complete, or nearly complete, genome sequence was present, we identified microorganisms with strong known association to CNS infections, such as *Enterovirus*, *Parvovirus B19*, and *Toxoplasma gondii*. Among microorganisms with complete or nearly complete genomes, the viruses were predominant compared with bacteria or other microorganisms. This may be due to centrifuging CSF prior to extraction, or alternatively, some reports have suggested that there is a greater sensitivity with metagenomics for viral agents when compared with other microorganisms (10–13). Full genomes of TTV (*n* = 8) and human pegivirus-1 (GBV-C; *n* = 6) were identified, yet a clear demonstration of their association with human CNS disease is still lacking. While some studies argue they may play a role in disease, the ubiquity of these viruses suggests they are commensals or constituents of the normal virome flora (14, 15). Their levels appear to increase in response to an infection but are unlikely to be the primary insult.

*Picobirnavirus* does not have a well-established association with CNS infection, nor has its pathogenicity been established. It has been detected in the stool of patients with gastroenteritis as well as in comparable specimens from several mammals and birds (16). More recently, picobirnavirus has been described in hospitalized patients with ARI in Uganda and Colombia (4, 17). In fact, the sequence recovered from the young boy in the present study branched within the very same genotype described in Berg et al. from Colombia and with related sequences from China and Cambodia causing ARI (4). We note that, 10 days before admission to the hospital, the mother indicated the child had an ARI. This is the first instance of *Picobirnavirus* associated with respiratory symptoms or neurologic involvement described in Brazil. *Picobirnavirus* potential neural pathogenicity described here agrees with a recent finding for a phylogenetically unrelated picobirnavirus causing encephalitis in Australia (18) (Figure 4A).

The presence of *Vesivirus* in 1 CSF sample was quite an unexpected observation. Vesiviruses are a genus in the *Caliciviridae* family, which infect a broad range of animals and can induce a variety of disease manifestations, including canine encephalitis (19). Vesiviruses have also been detected in dogs with diarrhea, glossitis, balanitis, or vesicular vaginitis (20, 21). Clinical manifestations in humans have been described only once, specifically from a laboratory worker with systemic illness, including vesicular lesions on all 4 extremities (22). To our knowledge, this is the first description of a CNS manifestation of *Vesivirus* in humans. Considering the ability of *Calicivirus* to cross the host species barrier (22) and the close social interactions between humans and dogs, it is essential to determine whether dogs harbor viruses such as these with zoonotic potential. Although further evidence is necessary to confirm that a host jump has occurred from canines, its detection in the current series is noteworthy.

Seven patients with *Enterovirus* infection were identified only by metagenomics who had previously tested negative for *Enterovirus* by commercial platforms and/or real time PCR tests. Four patients were determined to have *Enterovirus* A and 1 patient had *Enterovirus* B. One possible explanation for this is that the PCR methodology used (Film Array Biofire or Neuro 9 Biometrix) was not sensitive enough to identify the small amount of genetic material present in the samples. Another possibility would be the genetic diversity of these *Enterovirus* samples. As RNA viruses, enteroviruses are characterized by a great genetic variability relying on 2 different evolutionary mechanisms: mutation and recombination (23). Identifying specific strains missed by commercial tests should be valuable for improving diagnostics and informing surveillance and control measures against this disease. Although these *Enterovirus* infections were confirmed using an in-house PCR, the possibility that there still were inherent limitations of the previous commercial, PCR/prime/probe set cannot be ruled out. Previous studies analyzing other viral genomes have demonstrated that PCR assays relying on specific primers and probes may fail to detect all viral species, whereas metagenomic approaches have shown broader detection capabilities (24). Our previous study on the relation of the *Enterovirus* viral load to other laboratory parameters in CSF demonstrated that 30% of available instances of aseptic meningitis were associated with a low viral load (25).

Other microorganisms that we identified have been previously reported in CNS infections, such as *Mastadenovirus*, *Herpesvirus*, and *Polyomavirus* (1). Studies have detected *Bocavirus* by PCR in the CSF and linked these infections to encephalitis (26). Ad31 has also been implicated as a rare cause of encephalitis (27). On the other hand, microorganisms such as *Gemikibiviru*s, although previously identified in the CNS, do not yet have defined pathogenesis in CNS infections (28–31).

The limitations of our study must be acknowledged. First, the results presented were based on our interpretation of mNGS findings, which involved specific criteria and which may not be universally accepted. Alternative protocols for data normalization and interpretation of mNGS findings are possible. In agreement with previous suggestions, we believe that further studies are necessary to unify the threshold standards for defining mNGS positivity (32). Another limitation is the low number of reads above background that were detected for many microorganisms, although it should be remembered these were CSF samples that were negative for microorganisms upon initial testing. Thus, it would be expected that the level of any microbes present in these samples might be low and approach the limit of sensitivity for metagenomics. Even though many presumed infectious etiologies as identified by mNGS cannot be verified, the potential advantages of this technology are numerous. This diagnostic approach facilitates early diagnosis and treatment and enables the identification of emerging or previously unknown pathogens, coinfections, and an infectious etiology of undiagnosed diseases. Our data also support and confirm the utility of mNGS as a fundamental tool for early pathogen detection, prevention, and preparedness of future pandemics.

In conclusion, enteroviruses and herpesviruses, especially VZV, were the major causes of CNS infections in São Paulo, Brazil. The use of mNGS can identify potential pathogenic microorganisms in the CSF of individuals with acute meningitis or encephalitis that are not detected by standard diagnostic tests. The detection of a wide range of potential pathogens in CSF, identified only after metagenomic analysis, highlights the complexity of CNS infections and the potential of metagenomics to enhance diagnostic accuracy, inform treatment strategies, and assist in addressing future pandemics.

## Methods

## Sex as a biological variable

Both male and female participants in the study were included in this investigation. It was not considered as a biological variable in the metagenomic analysis.

### Study population

In this cross-sectional study, we retrospectively analyzed CSF samples from 600 patients with clinical suspicion of acute infectious encephalitis or meningitis enrolled in a hospital-based surveillance study conducted in São Paulo, Brazil, from March 2018 to November 2019. All patients were evaluated in 2 healthcare settings: (a) the Hospital das Clínicas da Faculdade de Medicina da Universidade de São Paulo (HC-FMUSP), a tertiary-level hospital (157 samples); or (b) emergency and community healthcare services in the city of São Paulo. These patients were screened by the Senne Liquor Laboratory (443 samples). All individuals were hospitalized and received proper care and treatment according to the results of the standard diagnostic tests performed and according to Brazilian guidelines.

### Inclusion criteria

Patients were initially interviewed and examined by an infectious disease physician or a neurologist who performed anamnesis, physical examination, and clinical data collection. After a clinical diagnosis of meningitis or encephalitis, a CSF sample was collected by lumbar puncture within 2–5 days from the onset of symptoms. Clinical diagnosis was designated in accordance with specific criteria for acute meningoencephalitis, as stated in current guidelines (33, 34).

### Diagnostic algorithm

As per the study protocol, all CSF samples underwent a complete cell count, differential leucocyte count, and biochemistry tests (including glucose, protein, and lactate concentrations) to analyze CSF parameters. For pathogen analysis, the following tests were performed: examination of Gram-stained and India Ink smears, bacterial and fungal cultures, and use of the BioFire FilmArray Meningitis/Encephalitis Panel (BioMérieux) and XGEN Multiplex Neuro 9 (Mobius Life).

The BIOFIRE ME Panel, for use with the BIOFIRE FILMARRAY systems, is a qualitative multiplex nucleic acid–based in vitro diagnostic test capable of the simultaneous detection and identification of nucleic acids from bacteria (*E. coli K1*, *Haemophilus influenzae*, *Listeria monocytogenes*, *Neisseria meningitidis*, *Streptococcus agalactiae*, *Streptococcus pneumoniae*), viruses (*Cytomegalovirus*, *Enterovirus*, herpes simplex virus 1, herpes simplex virus 2, HHV-6, human parechovirus, VZV), and fungal (*C*. *neoformans/C*. *gattii*). 

The XGEN MULTI N9 is another qualitative multiplex nucleic acid-based in vitro diagnostic test, capable of the simultaneous detection and identification of nucleic acids from different viruses, including human adenovirus, EBV, herpes simplex virus 1 and herpes simplex virus 2, and VZV.

At the discretion of the attending physician, supplementary tests were performed on the CSF, such as tuberculosis culture, PCR for toxoplasmosis, and immunologic tests for syphilis.

After this routine evaluation, samples were immediately stored at 4°C and shipped to the laboratory within 24 hours. At the Virology Laboratory in the Tropical Medicine Institute at Sao Paulo University Medical School, the CSF samples were stored at −80°C until tested.

Samples that tested negative during the initial screening, which included microbiological and molecular tests (Film Array Meningitis/Encephalitis Panel and the XGEN Multiplex Neuro 9 Panel), were again tested at the Virology Laboratory. A combination of real-time PCR tests was designed to identify viral and bacterial agents: *Enterovirus*, *Herpesvirus*, *Parechovirus*, *Influenzavirus A/B*, mumps virus, measles virus, HIV, *Polyomavirus* (JC virus [JCV] and BKV), *Mastadenovirus adami* (early named human adenovirus), *Parvovirus* B19 and principal arboviruses, such as dengue virus (1–4), chikungunya virus, Zika virus, yellow fever virus, Mayaro virus, West Nile virus, and *Listeria* (35–50).

### Metagenomic assessment

Briefly, 500 μL of CSF was centrifuged at 12,000*g* for 10 minutes. Then, 200 μL of the supernatant was automatically extracted for total nucleic acids (DNA and RNA) using the Extracta 96 Fast Kit (Loccus). cDNA synthesis was performed using random decamer primers and Superscript IV reverse transcriptase, according to the manufacturer’s protocol (Thermo Fisher Scientific). Second-strand cDNA synthesis was carried out using DNA Polymerase I Large Fragment (Thermo Fisher Scientific). In the final process, double-stranded DNA was obtained and quantified using the QuantiFluor ONE dsDNA System (Promega) in Quantus Fluorometer (Promega).

For library preparation, 2 ng of double-stranded DNA in 5 μL of the total nucleic acid was input for the Nextera XT kit (Illumina), following the manufacturer’s instructions. Purification and size selection were performed following the double-sided bead purification method with ProNex Size Selective Purification System (Promega). The quantity of each sample was normalized using the ProNex NGS Library Quant Kit (Promega) to ensure equal representation of the library within the pooled library. The pool underwent size selection, targeting a 400 bp insert (range, 400–700 bp) using Pippin Prep (Sage Science) to exclude very short or long fragments. Library quality was assessed with an Agilent 2100 Bioanalyzer (Agilent Technologies) using a high-sensitivity DNA kit. The library was prepared by pooling a 10 nM concentration of purified pool for sequencing on an Illumina NovaSeq 6000 sequencer using a 500-cycle paired-end sequencing strategy.

FASTQ files were evaluated using DiVir 3.0, a proprietary bioinformatics pipeline developed at Abbott, which trims adapters and removes low-quality reads and then uses BWA 0.7.18 (51), BLAST 2.16.0, and MMSeqs2 17-b804f (52) to taxonomically classify remaining reads as either background, human, prokaryotic, fungal, plant, invertebrate, or viral. Contigs were generated using SPAdes 4.0.0 (53). Sequencing reads were normalized based on total reads and subtracting against a nontemplate control (NTC) to enable comparison of samples with varying sequencing depths.

### Interpretation of mNGS results for microbial infection

#### Exclusion of contaminants.

An analysis was conducted as described to ignore likely contaminants in samples and reduce the background noise typically observed in shotgun metagenomics studies (54, 55). Sodium hypochlorite and water were used as negative controls. Several microorganisms have been recognized in previous studies as contaminants in mNGS analyses (55). Based on these findings, along with their detection in negative control samples in the present investigation, the presence of *Novosphingobium sp.*, *Cutibacterium* sp., *Sphingomonas* sp., *Brachybacterium* sp., and *Eukarya* such as *Malassezia* sp., *Cryptocaryon* sp*.*, *Parastagonospora* sp*.*, *Pinus* sp*.*, *Musa sp.*, *Haplochthonius* sp*.*, *Coniosporium* sp*.*, *Panicum* sp*.*, *Drosophila* sp*.*, *Exserohilum* sp*.*, *Diaphorina* sp*.*, *Pseudomicrostroma* sp*.*, *Culex* sp*.*, *Triticum* sp., or *Elaeis* sp. when detected in any sample were excluded from our final analysis (55).

#### Graphical representation of data.

The host range of mapped viruses was determined using species names from NCBI. Viruses associated with bacteria (bacteriophages) were filtered out based on their host information. The remaining viruses were then parsed into NCBI taxonomy files by genus name using the taxonomizer package in RStudio (https://cran.r-project.org/web/packages/taxonomizr/index.html). Only samples with identified eukaryotic viruses were included in this study. Subsequently, the number of reads assigned to viral families and species was converted into relative values (reads per million [RPM]) to estimate their abundance within each sample. Genome composition data were retrieved from ICTV based on virus genus taxonomy.

All statistical analyses were performed using R version 4.0.1 within RStudio version 1.3.959. Graphs were generated using the Complex Heatmap (56) and ggplot2 (57) packages in R and Kronaplot (58) to explore taxonomy and relative abundance of eukaryotic viruses.

#### Group definitions.

An individual was considered positive for any virus or any bacterial or Eukaria taxon when the agent was represented by their complete genome or by partial sequences comprising more than 10% of the genome and spanning 3 distinct regions. The microorganisms remaining after applying this selection criteria were classified into 3 distinct groups: (a) group 1, which represents all samples in which a complete genome or a near-full length genomic sequence for 1 or more microorganisms was identified; (b) group 2, which represents all samples in which partial sequences were identified comprising more than 10% of the genome across 3 distinct regions; and (c) group 3, which represents undefined instances, or all identified microorganisms not included in groups 1 or 2. 

### Statistics

*P* values of less than 0.05 were considered significant.

### Study approval

This study was approved by the local ethics committee (Comissão Nacional de Ética em Pesquisa [CONEP]), protocol no. CAAE: 67203417.0.0000.0068. Informed consent was obtained from all patients or patients’ parents.

### Data availability

Data sets related to this article can be found in National Center for Biotechnology Information’s BioProject with submission identification PRJNA1224245 (https://www.ncbi.nlm.nih.gov/bioproject/PRJNA1224245), and the list accompanied by the individual descriptions of the samples can be found in Supplemental Table 3. GenBank Nonredundant (NR) accession numbers include: PV203681 (Picobirnavirus, https://www.ncbi.nlm.nih.gov/nuccore/PV203681) and PV203682 (Vesivirus, https://www.ncbi.nlm.nih.gov/nuccore/PV203682). Values for all data points in graphs are reported in the Supporting Data Values file.

## Author contributions

MCMC, NEF, and ACDC designed research studies; NEF, LH, and MCMC conducted experiments; NEF, MLMM, HGOP, CGTS, ACSDO, and MVT acquired data; NEF, ACDC, MFC, NEF, ACDC, TRTM, and MCMC analyzed data; EGK, MCMC, GAC, MGB, HRG, and MAR provided reagents; TRTM, SSW, MCMC, NEF, GDOR, RBD, CS, NEF, and MGB wrote the manuscript; and all authors have read and agreed to the published version of the manuscript.

## Supplementary Material

Supplemental data

ICMJE disclosure forms

Supporting data values

## Figures and Tables

**Table 1:**
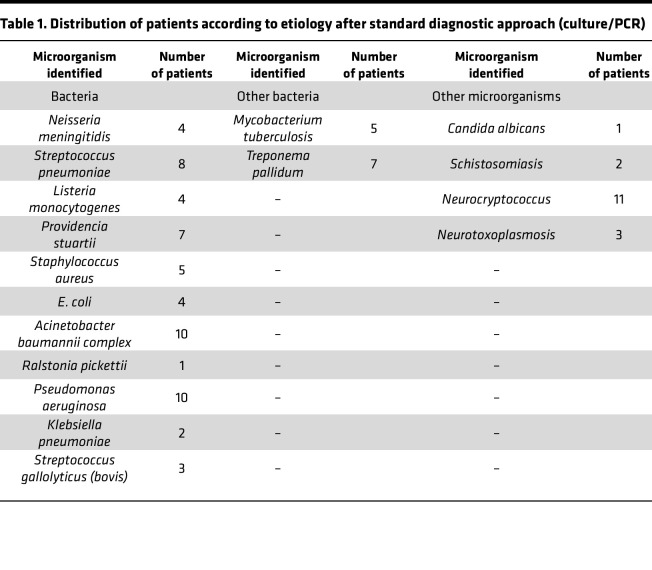
Distribution of patients according to etiology after standard diagnostic approach (culture/PCR)

**Table 2:**
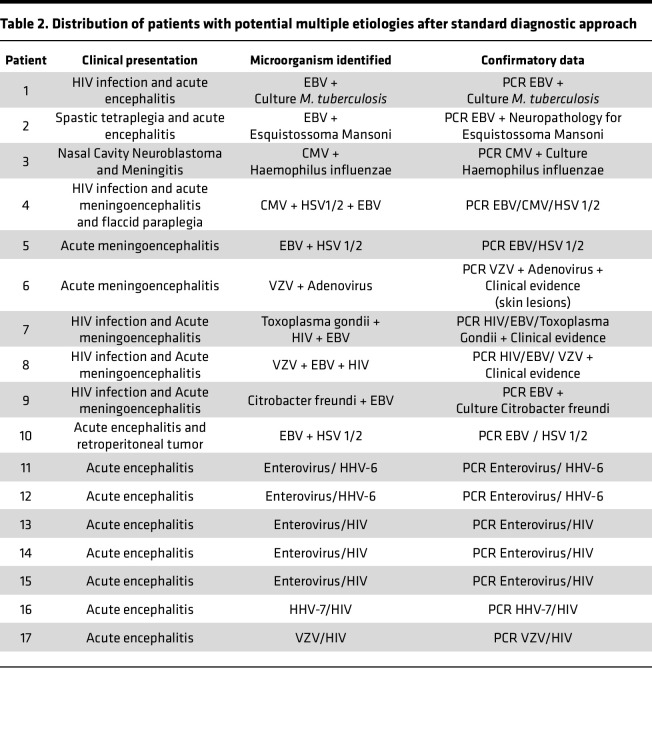
Distribution of patients with potential multiple etiologies after standard diagnostic approach

**Table 3:**
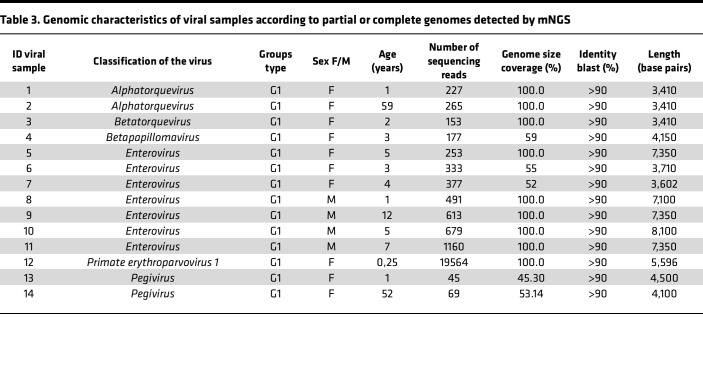
Genomic characteristics of viral samples according to partial or complete genomes detected by mNGS

## Version 1 05/20/2025

In-Press Preview

## Version 2 07/08/2025

Electronic publication
