# Supplementary material for: Metagenomic detection of central nervous system infections missedby conventional testing
Source: JCI Insight. 2025 May 20;10(13):e189295. doi: 10.1172/jci.insight.189295 (PMC12306619; doi:10.1172/jci.insight.189295)
Supplement: Supplemental data [file jciinsight-10-189295-s240.pdf]

Table 1 S (Supplementary material): Data of 230 sequenced samples pre- and post-filtering metrics, highlighting improvements in data quality applying the Host filtration, adapters remove, remove duplicate reads, low complexity sequences, low quality bases and short reads (< 35 bp)

Sample ID	Total Reads	Quality Control	Passed filters (%	GRCh38 (%)	sert size media	Archea	Bacteria	Eukarya	Phage	Viruses
57065	17690148	4121776	23.3	35	219	46	3074669	256509	5030	3316
59950	9074032	70250	0.774	83	207	1	41174	4053	88	15
59993	11384124	185640	1.631	80	213	0	128630	5649	123	38
59996	15454006	2020308	13.073	57	218	16	1486256	52620	1285	163
60371	14118168	4683440	33.173	24	218	31	3852956	298506	1879	225
60376	14215974	1401886	9.861	58	213	54	932805	140297	1108	318
60581	9165006	1134204	12.375	45	221	136	717457	57622	814	103
60688	18062982	58774	0.325	82	221	2	42320	4442	60	14
60690	8089142	182	0.002	84	204	0	97	13	2	2
60906	12412402	68050	0.548	88	213	0	47110	7454	79	47
60907	11252410	1279204	11.368	55	213	20	853151	117201	971	184
60920	16848606	55490	0.329	85	204	2	36859	1226	70	11
60940	9502670	992344	10.443	55	213	81	621257	45951	839	161
60999	17824216	7084236	39.745	15	216	51	5601550	472239	1880	340
61039	11108940	4395458	39.567	8	209	288	3461566	165617	1430	149
61041	9772780	2571606	26.314	19	207	103	1613156	167831	1159	195
61089	7493776	7078	0.094	85	208	0	5175	449	35	29
61122	12108322	2708000	22.365	29	208	61	1928849	97922	1575	121
61147	8998716	140594	1.562	80	213	2	85163	2186	17	6
61170	14800432	1422750	9.613	59	218	4	988717	72610	1241	336
61200	17173418	669866	3.901	71	222	7	477707	30740	754	84
61236	13443838	770696	5.733	74	216	7	531425	23269	620	162
61258	16710174	4902150	29.336	21	217	70	3621172	382579	3820	581
61335	10604530	6054	0.057	79	196	0	4352	548	9	0
61493	4041182	65900	1.631	88	85	3	22182	603	32	5
61499	3425336	13800	0.403	84	77	1	4003	210	9	0
61510	8694560	109482	1.259	83	205	5	64253	3900	77	15
61528	12532808	1736134	13.853	44	212	49	1197427	59308	1073	388
61529	3320628	152304	4.587	71	134	7	73767	3365	32	11
61766	3576810	21034	0.588	86	148	5	8896	168	16	0
61771	2636242	67254	2.551	80	111	0	28467	1596	45	14
61806	10968408	80720	0.736	82	201	1	60638	2189	72	34
61866	3727070	26658	0.715	86	136	0	11469	199	6	34
61867	3721810	43174	1.16	84	185	2	20406	180	8	8
61880	4154866	3150	0.076	89	154	2	1076	42	0	2
61968	16051344	1013748	6.316	71	219	31	708181	43212	1127	209
61977	3906334	7020	0.18	95	209	0	2619	57	8	340
62086	11169306	236538	2.118	81	213	9	157127	12356	177	27
62384	5353276	8848	0.165	85	132	2	3054	36	1	0
62655	9337438	109954	1.178	84	213	9	70388	4830	138	10
62760	2580946	30086	1.166	84	183	4	15265	188	0	0
62764	5485170	164980	3.008	76	174	4	87821	2100	21	34
62765	4386108	103090	2.35	81	128	3	37379	307	35	8
62768	3416174	376388	11.018	48	157	17	152856	2351	81	23
62770	14084660	1504754	10.684	45	205	64	1045968	24247	572	60

62771	7362168	774	0.011	84	182	0	204	12	0	60
62805	2408134	27786	1.154	81	174	0	11100	947	28	44
62836	9785936	13382	0.137	89	228	0	9610	551	24	6
62837	2569642	61030	2.375	80	156	0	25714	609	232	16
62839	4383676	30102	0.687	80	91	0	13742	323	51	4
62841	2253204	68386	3.035	67	117	0	28368	581	209	30
62846	3498016	21236	0.607	85	121	0	7169	262	49	2
62849	4473826	17992	0.402	82	174	4	7695	171	29	64
62868	2195238	13360	0.609	83	190	0	9630	588	6	0
62870	4049276	42738	1.055	79	108	2	16102	338	12	8
62907	16747718	3124966	18.659	30	216	10	2409901	78475	1413	252
62919	20506706	2763800	13.478	53	216	236	1861353	126315	3706	774
63102	6113990	5962	0.098	86	156	0	2030	18	0	2
63104	6433894	5696	0.089	82	189	0	2161	25	0	2
63105	5978894	1166706	19.514	30	167	17	526456	254451	92	57
63107	8269760	1614	0.02	83	202	0	1199	2	0	0
63111	1734930	4256	0.245	89	98	0	1558	50	8	10
63112	5865220	1126	0.019	87	171	0	383	10	1	0
63115	4161368	30780	0.74	84	167	0	17974	276	8	2
63118	2264500	25346	1.119	81	155	1	9999	213	23	12
63120	5222144	124728	2.388	82	177	0	74773	6765	121	8
63185	4878660	6556	0.134	85	111	0	2635	125	29	0
63187	5922366	1085854	18.335	28	192	20	695593	29950	407	850
63188	6605630	3774	0.057	86	168	0	1389	23	0	6
63189	15318404	1720412	11.231	62	221	42	1316483	80271	1203	3783
63190	3199948	64886	2.028	83	172	3	28098	245	13	6
63191	3668060	29482	0.804	83	136	2	10280	240	4	9
63195	476874	33428	7.01	9	78	4	14711	879	24	45
63196	3409078	175874	5.159	79	90	9	74978	1175	71	69
63200	7739084	1072590	13.859	48	206	12	658325	77596	1227	106
63203	5081672	5722	0.113	91	201	0	2812	56	2	0
63207	3322710	121428	3.654	71	209	2	83739	2924	78	12
63293	6060776	319584	5.273	64	196	4	195973	1537	73	6
63294	5067232	27352	0.54	84	108	4	10263	132	12	28
63296	6484382	18142	0.28	86	95	0	9751	176	7	2
63297	2141934	72992	3.408	74	122	6	30333	1089	27	332
63298	3681262	10276	0.279	82	156	0	4418	24	2	38
63299	4102862	115236	2.809	81	136	2	48114	710	27	31
63301	6543952	58304	0.891	85	178	2	30651	396	1	10
63303	3032702	25604	0.844	81	133	0	10603	287	2	2
63305	5094606	418580	8.216	70	166	14	203272	1868	243	60
63307	4860674	57086	1.174	86	112	1	21561	434	41	18
63308	4485938	11794	0.263	91	133	0	4119	495	12	0
63311	4179560	72256	1.729	83	137	4	29444	413	57	14
63403	991428	162592	16.4	20	171	11	79071	1162	30	1156
63405	4872228	860748	17.666	40	157	20	421053	5341	95	956
63407	4873048	17002	0.349	86	123	0	6892	352	9	0
63408	2626614	228984	8.718	61	153	7	106666	1627	65	36

63409	2936724	19524	0.665	83	135	0	8141	265	29	16
63410	2473092	655180	26.492	24	174	44	396620	15696	117	314
63412	9656394	29422	0.305	86	213	0	18019	848	21	23
63468	5461464	864632	15.832	14	230	76	553494	2709	35	218
63469	5406910	68520	1.267	84	200	0	40777	786	16	10
63471	6494630	27836	0.429	85	130	2	11566	157	7	80
63472	4467602	18112	0.405	86	174	0	7771	204	1	2
63473	5333726	202834	3.803	73	186	25	107459	8612	84	18
63474	4810582	11168	0.232	89	118	0	4620	122	0	0
63476	3454672	18540	0.537	78	137	0	7597	63	0	0
63480	5302172	4876	0.092	88	158	0	1785	26	0	80
63570	12575852	529194	4.208	72	213	22	290684	15214	276	248
63574	10820946	133998	1.238	84	227	2	88979	10205	87	74
63602	2380328	12406	0.521	81	126	0	4889	313	5	277
63608	9799812	8928	0.091	86	218	1	5108	649	16	18
63612	5832052	17022	0.292	89	114	2	5567	117	5	0
63618	2258800	136544	6.045	54	142	21	54189	2087	78	13
63628	4211772	12984	0.308	88	176	2	5623	308	8	2
63660	8988984	182090	2.026	85	237	0	2959	49	8	175861
63687	4482098	268878	5.999	63	173	16	140843	1502	24	32
63692	3576226	52774	1.476	79	173	2	21938	591	73	6
63694	2655430	39990	1.506	89	168	0	16607	575	53	6
63701	8210968	22598	0.275	85	219	0	17518	1074	29	40
63702	7762728	2464	0.032	82	219	0	1681	22	0	4
63702	5248752	2176	0.041	92	182	0	846	23	0	4
63702	5248752	2176	0.041	92	182	0	848	97	8	2
63705	4486634	128770	2.87	80	185	8	65315	1078	171	12
63728	3286570	54314	1.653	88	176	1	25033	981	12	4
63732	4242248	8612	0.203	95	211	1	4630	99	3	4
63733	8032184	106008	1.32	83	199	1	61965	7173	62	12
63735	5436554	6670	0.123	87	131	0	2532	60	0	0
63738	4650482	4206	0.09	88	159	2	1484	52	0	0
63739	2210030	105962	4.795	72	154	1	57353	6479	95	2
63741	4673568	19334	0.414	87	122	0	10501	199	18	6
63742	2891456	66694	2.307	84	101	1	27484	3596	44	59
63745	4037086	14656	0.363	91	113	0	6051	168	11	22
63767	9509886	47866	0.503	85	206	2	26435	3373	29	2
63770	3999608	311124	7.779	73	169	25	191657	9105	265	74
63773	4564708	3882	0.085	93	172	0	1913	16	6	0
63775	5937784	82504	1.389	80	191	2	50562	3038	174	14
63777	8208908	53462	0.651	87	215	0	36199	1576	22	24
63779	8359324	106596	1.275	81	207	2	63521	1992	15	58
63780	24467804	3224418	13.178	36	220	125	2074173	58996	406	2139
63783	9081814	771916	8.5	53	214	27	492983	11313	409	139
63800	12442644	90792	0.73	85	213	4	55867	2430	163	102
63801	7613634	547008	7.185	71	214	4	302857	11280	262	75
63805	11971046	843482	7.046	70	213	17	483857	136075	363	118
63807	6520444	10700	0.164	89	210	2	5899	361	39	6

63809	6216768	13934	0.224	90	241	0	5546	2201	113	14
63810	4959834	1066902	21.511	29	230	10	695946	45457	1638	1563
63813	12593676	88986	0.707	90	243	0	53323	2955	248	160
63818	12868664	2106760	16.371	21	222	99	1277614	168030	194	320
63831	13186772	1636	0.012	83	243	0	952	34	0	0
63833	11646686	306750	2.634	75	218	21	175541	24438	27	25
63836	8084626	29366	0.363	86	215	12	16308	110	32	6
63838	11027402	87812	0.796	83	227	0	54850	1072	20	53
63840	5265800	709236	13.469	33	214	0	469093	5997	244	67
63850	18055880	353196	1.956	84	240	0	204381	3959	858	417
63851	12462472	174104	1.397	89	228	7	102413	4747	487	63
63852	7864772	5366	0.068	88	229	0	2920	51	0	0
63854	6596940	35446	0.537	84	194	3	19369	355	5	0
63855	9599970	433104	4.512	68	217	12	260538	5770	89	330
63859	9701254	345274	3.559	70	211	6	209726	3473	11	16
63860	9470650	416466	4.397	67	218	6	255457	4350	1530	98
63899	5881344	5840	0.099	85	244	0	1464	16	79	2898
63902	10859992	1181350	10.878	47	206	1	702594	10467	1129	372
63903	14976848	125426	0.837	92	258	1	101545	3857	134	19
63912	7648516	3646	0.048	88	235	0	1879	24	0	2
63913	11257586	64852	0.576	85	223	3	31190	244	2	0
63931	10716904	1866532	17.417	28	217	25	1167793	105216	2229	332
63934	8421070	624	0.007	86	225	0	330	1	0	18
63935	7710832	12702	0.165	87	214	0	6447	58	4	144
63937	10908514	47870	0.439	85	225	0	26002	527	44	6
63939	12579560	41436	0.329	87	229	2	23866	246	121	25
63940	9123724	20078	0.22	85	216	0	10855	86	36	10
63941	11533284	479516	4.158	79	256	0	259942	43171	732	70
63943	7457916	131984	1.77	85	223	0	56580	905	602	28
63944	9274024	69136	0.745	84	207	0	47601	2056	53	6
63945	8358296	37394	0.447	82	203	8	17408	1017	7	3
63946	11097704	12534	0.113	87	234	0	6930	137	4	0
63965	8538770	252524	2.957	74	214	2	159368	1446	32	14
63967	9087250	3464	0.038	85	216	0	1915	208	10	2
63968	5521556	1854	0.034	87	212	0	857	18	0	0
63969	9717988	655604	6.746	54	215	118	416806	2680	868	151
63975	9485762	417132	4.397	64	226	5	246764	2635	571	110
63976	4497056	406212	9.033	50	216	2	236845	3816	847	48
63996	4015080	722894	18.004	12	221	9	454330	4388	1183	83
63997	5413876	929860	17.175	7	236	2	484847	7252	4328	735
63998	68364	3404	4.979	53	187	0	1611	21	4	4
63999	7753888	24844	0.32	84	198	6	14764	379	8	0
64001	7864928	2238	0.028	89	201	0	880	44	0	2
64002	9936382	6938	0.07	88	224	0	3303	96	0	38
64004	8859260	42482	0.48	82	213	0	25578	569	5	4
64005	9490562	63478	0.669	81	211	2	30739	1471	105	19
64007	15231826	13288	0.087	84	222	1	5672	888	46	12
64008	8912778	337376	3.785	79	208	4	33881	55058	66	20

64009	8095434	1897342	23.437	18	235	2	1209192	79367	9786	3717
64011	13956602	390498	2.798	78	224	7	223603	25029	515	362
64012	7227356	1164970	16.119	40	221	5	778626	25471	6395	505
64013	2762982	174490	6.315	71	185	33	82328	3375	149	14
64034	10292638	517090	5.024	77	245	161	317982	48353	729	583
64036	11836090	30698	0.259	86	226	1	17164	191	8	506
64037	1907344	108860	5.707	64	210	4	67435	671	2	6
64038	14468890	1438488	9.942	44	220	24	989806	8707	2048	168
64039	12505616	40450	0.323	82	220	0	24448	561	42	14
64041	5774660	522326	9.045	53	217	14	340252	8654	573	93
64042	8427820	121678	1.444	81	231	3	75651	1439	198	45
64045	15274822	970798	6.356	58	216	23	621793	7477	1117	443
64046	19920082	7514122	37.721	22	230	6026	5298862	88544	5370	1415
64047	3864932	44446	1.15	90	185	5	29190	1584	86	29
64050	3956750	542390	13.708	52	205	22	385393	29159	298	119
64091	12511340	271802	2.172	79	227	6	199120	5332	123	24
64093	4387592	44446	1.013	82	205	0	25445	366	2	315
64094	12362052	40762	0.33	86	229	0	24848	575	52	16
64096	8763250	15136	0.173	82	210	0	7916	477	20	4
64098	11664074	82156	0.704	81	211	3	51565	3034	92	17
64099	20124826	2621198	13.025	48	240	131	1869332	39753	11954	1184
64101	17065992	66292	0.388	82	222	1	44180	873	102	32
64102	12798974	554644	4.334	74	216	62	276157	15958	1753	214
64104	49828	1982	3.978	70	175	0	1483	36	4	0
64105	9480650	1867910	19.702	53	217	19	1448686	74956	1373	415
64108	13764670	147654	1.073	79	203	13	98458	6640	64	23
64109	9919724	27104	0.273	85	219	0	14910	1972	5	26
64113	9503550	48332	0.509	84	206	0	32224	1579	52	14
64115	11252440	57822	0.514	82	202	5	37431	2672	37	14
64116	11120716	105338	0.947	84	218	4	71202	5280	90	12
64120	6532970	6236	0.095	89	212	0	4439	275	15	0
64121	9339686	32774	0.351	86	210	0	21019	1579	18	0
64122	10247970	112424	1.097	88	210	5	76356	4124	52	16
64124	11220512	1160394	10.342	56	198	5	875025	32913	547	93
64153	11359156	4160	0.037	89	220	0	1994	111	20	0
64156	21258742	3012138	14.169	41	212	42	2135784	55454	2077	345
64159	22812814	1965064	8.614	63	221	77	1325610	61732	2478	417
64161	10133188	8666	0.086	80	214	0	7158	601	47	0
64164	9832010	1441136	14.658	45	204	38	856572	148828	1122	309
64166	8069854	1797392	22.273	41	211	7	1494354	45525	808	136
64167	14654426	1509206	10.299	52	202	25	1091367	47840	1197	9210
Undetermined	109806734	2698670	2.458	54	292	202	1707017	89399	3678	31975

Table 2 S (Supplementary material) - Confirmatory tests -Sequence alignment of primers and probes

Type	Forward (3'-5')	Reverse (3'-5')	Probe	Reference
Enterovirus sp	CCCTGAATGCGGCTAATCC	ATTGTCACCATAAGCAGCCA	AACCGACTACTTTGGGTGTCCGTGTTTC	Verstrepen, W.A .et. al. J Clin Microbiol. 2001
Primate erythroparvovirus 1	TGCAGATGCCCTCCACCCA	GCTGCTTTCACTGAGTTCTTC	ACCTCCAAACCACCCCAATTGTCACA	Alves, A.D.R. et. al. Expert Review of Molecular Diagnostics, 2019.
Torque Teno virus sp	AGCCCGGCCAGTCC	GTGCCGIAGGTGAGTTTA	TCAAGGGGCAATTCGGGCT	Maggi, F. et. al. J Med Virol. 2001
HIV-1 (A,G)	COD: 4448892 - NCM: 38220090 / TAQMan/ MAO Genica			Applied BioSystems® Waltham, EUA
Pegivirus	TTACGACGACTGCCCTTACA	ACAGTGTTTCCCGGCACAT	AAAAGTICGCGGCGTCAA	PhD Thesis https://doi.org/10.11606/T.5.2019.tde-16042019-113655
Picobirnavirus	1Round CCGCAACTATAGGTGGCAAGG<br>2Round TAAGATGGCGGCCATACTTGG	1Round CCGCGTCATCTTCACCCTTGG<br>2Round GATCTTTTCGTCTACCTCTTCC	-	Virology Laboratory IMT USP

Table 3 S (supplementary). List accompanied by the individual

Run accession	Biosample accession	Patient ID
SRR32359116	SAMN46854210	57065
SRR32359115	SAMN46854211	59993
SRR32359101	SAMN46854212	60371
SRR32359090	SAMN46854213	60376
SRR32359079	SAMN46854214	60581
SRR32359068	SAMN46854215	60906
SRR32359057	SAMN46854216	60907
SRR32359046	SAMN46854217	60940
SRR32359035	SAMN46854218	60999
SRR32359024	SAMN46854219	61039
SRR32359114	SAMN46854220	61041
SRR32359110	SAMN46854221	61122
SRR32359109	SAMN46854222	61147
SRR32359108	SAMN46854223	61170
SRR32359107	SAMN46854224	61200
SRR32359106	SAMN46854225	61236
SRR32359105	SAMN46854226	61528
SRR32359104	SAMN46854227	61771
SRR32359103	SAMN46854228	61866
SRR32359102	SAMN46854229	61867
SRR32359100	SAMN46854230	61968
SRR32359099	SAMN46854231	61977
SRR32359098	SAMN46854232	62764
SRR32359097	SAMN46854233	62768
SRR32359096	SAMN46854234	62771
SRR32359095	SAMN46854235	62805
SRR32359094	SAMN46854236	62841
SRR32359093	SAMN46854237	62849
SRR32359092	SAMN46854238	62870
SRR32359091	SAMN46854239	62907
SRR32359089	SAMN46854240	62919
SRR32359088	SAMN46854241	63105
SRR32359087	SAMN46854242	63111
SRR32359086	SAMN46854243	63118
SRR32359085	SAMN46854244	63187
SRR32359084	SAMN46854245	63189
SRR32359083	SAMN46854246	63191
SRR32359082	SAMN46854247	63195
SRR32359081	SAMN46854248	63200
SRR32359080	SAMN46854249	63207
SRR32359078	SAMN46854250	63294
SRR32359077	SAMN46854251	63297
SRR32359076	SAMN46854252	63298

SRR32359075	SAMN46854253	63299
SRR32359074	SAMN46854254	63305
SRR32359073	SAMN46854255	63307
SRR32359072	SAMN46854256	63403
SRR32359071	SAMN46854257	63405
SRR32359070	SAMN46854258	63408
SRR32359069	SAMN46854259	63409
SRR32359067	SAMN46854260	63410
SRR32359066	SAMN46854261	63468
SRR32359065	SAMN46854262	63469
SRR32359064	SAMN46854263	63471
SRR32359063	SAMN46854264	63480
SRR32359062	SAMN46854265	63570
SRR32359061	SAMN46854266	63602
SRR32359060	SAMN46854267	63608
SRR32359059	SAMN46854268	63618
SRR32359058	SAMN46854269	63660
SRR32359056	SAMN46854270	63701
SRR32359055	SAMN46854271	63742
SRR32359054	SAMN46854272	63745
SRR32359053	SAMN46854273	63770
SRR32359052	SAMN46854274	63779
SRR32359051	SAMN46854275	63780
SRR32359050	SAMN46854276	63783
SRR32359049	SAMN46854277	63800
SRR32359048	SAMN46854278	63810
SRR32359047	SAMN46854279	63818
SRR32359045	SAMN46854280	63840
SRR32359044	SAMN46854281	63850
SRR32359043	SAMN46854282	63855
SRR32359042	SAMN46854283	63860
SRR32359041	SAMN46854284	63899
SRR32359040	SAMN46854285	63902
SRR32359039	SAMN46854286	63931
SRR32359038	SAMN46854287	63969
SRR32359037	SAMN46854288	63976
SRR32359036	SAMN46854289	63997
SRR32359034	SAMN46854290	63998
SRR32359033	SAMN46854291	64002
SRR32359032	SAMN46854292	64005
SRR32359031	SAMN46854293	64009
SRR32359030	SAMN46854294	64011
SRR32359029	SAMN46854295	64012
SRR32359028	SAMN46854296	64013
SRR32359027	SAMN46854297	64034

SRR32359026	SAMN46854298	64036
SRR32359025	SAMN46854299	64037
SRR32359023	SAMN46854300	64045
SRR32359022	SAMN46854301	64046
SRR32359021	SAMN46854302	64047
SRR32359020	SAMN46854303	64050
SRR32359019	SAMN46854304	64093
SRR32359018	SAMN46854305	64099
SRR32359017	SAMN46854306	64102
SRR32359016	SAMN46854307	64105
SRR32359015	SAMN46854308	64156
SRR32359014	SAMN46854309	64159
SRR32359113	SAMN46854310	64164
SRR32359112	SAMN46854311	64166
SRR32359111	SAMN46854312	64167

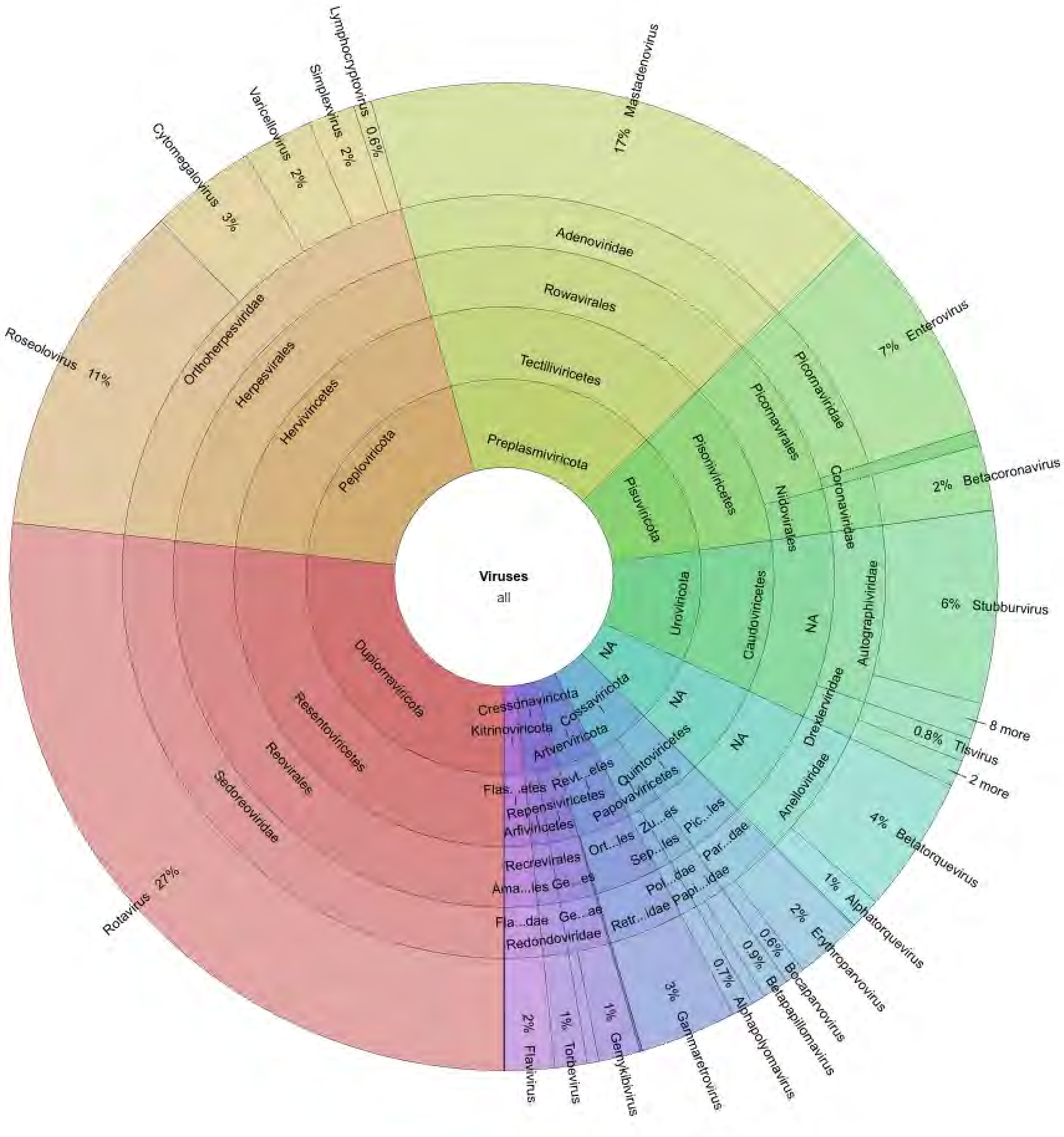

Viruses
all
17% Mastadenovirus
Lymphocryptovirus 0.6%
Simplexvirus 2%
Varicellovirus 2%
Cytomegalovirus 3%
Roseolovirus 11%
Adenoviridae
Rowavirales
Tectiliviricetes
Preplasmiviricota
Orthoherpesviridae
Herpesvirales
Herviviricetes
Peploviricota
7% Enterovirus
Picornaviridae
Picornavirales
Pisoniviricetes
Pisuviricota
Nidovirales
Coronaviridae
2% Betacoronavirus
6% Stubburvirus
Autographiviridae
NA
Caudoviricetes
Uroviricota
Drexlerviridae
8 more
0.8% Tlsvirus
2 more
Anelloviridae
4% Betatorquevirus
1% Alphatorquevirus
2% Erythroparvovirus
0.6% Bocaparvovirus
0.9% Betapapillomavirus
0.7% Alphapolyomavirus
3% Gammaretrovirus
1% Gemykibivirus
1% Torbevirus
2% Flavivirus
Rotavirus 27%
Sedoreoviridae
Reovirales
Resentoviricetes
Duplornaviricota
Cressdnaviricota
Kitrinoviricota
Cossaviricota
Artverviricota
Quintoviricetes
Papovaviricetes
Flas...etes
Revt...eles
Repensiviricetes
Arfiviricetes
Recrevirales
Ama...les
Ge...es
Ort...les
Zu...es
Pic...les
Sep...les
Par...dae
Pol...dae
Papi...idae
Retr...idae
Fla...dae
Ge...ae
Redondoviridae

Supplemental Figure 1. Pie chart depicting the relative abundance of microbial taxa from a metagenomic dataset. The color gradients represent different taxonomic groups, with the size of each segment corresponding to the relative abundance of that taxon in the sample.
